# Exploring the Microbiota of Diabetic Foot Infections With Culturomics

**DOI:** 10.3389/fcimb.2018.00282

**Published:** 2018-08-14

**Authors:** Joanne Jneid, Nadim Cassir, Sophie Schuldiner, Nathalie Jourdan, Albert Sotto, Jean-Philippe Lavigne, Bernard La Scola

**Affiliations:** ^1^MEPHI, APHM, IRD, Aix Marseille University, IHU-Méditerranée Infection, Marseille, France; ^2^Service des Maladies Métaboliques et Endocriniennes, Centre Hospitalo-Universitaire de Nîmes, Nîmes, France; ^3^Service des Maladies Métaboliques et Endocriniennes, Centre Hospitalo-Universitaire Carémeau, Nîmes, France; ^4^Service des Maladies Infectieuses et Tropicales, Centre Hospitalo-Universitaire Carémeau, Nîmes, France; ^5^INSERM, Université de Montpellier, Nîmes, France; ^6^Service de Microbiologie, Centre Hospitalo-Universitaire Carémeau, Nîmes, France

**Keywords:** microbiota, foot infection, diabetes, culturomics, bacterial species

## Abstract

The purpose of this prospective observational study was to evaluate the richness and diversity of bacteria in samples from diabetic foot infections using a culturomics approach. Bacterial culture findings from wound samples were analyzed together with clinical characteristics and treatment outcomes. We included 43 patients admitted to a French referral center with a moderate to severe diabetic foot infection. The 30,000 colonies identified yielded 53 different bacterial species. The global α-Shannon diversity was 3.34 and the bacterial richness per patient was 4 ± 2. Of all the identified bacterial species, 19 (35.8%) had never been previously cultured or identified by molecular methods from diabetic foot ulcers. Most of the samples were polymicrobial (*N* = 38; 88.3%). Of all the isolated species, the most prevalent were *Staphylococcus aureus* (*N* = 28; 52.8%), *Enterococcus faecalis* (*N* = 24; 45.2%), *Enterobacter cloacae* (*N* = 12; 22.6%), *Staphylococcus lugdunensis* (*N* = 10; 18.7%), *Staphylococcus epidermidis* (*N* = 6; 11.3%), *Proteus mirabilis* (*N* = 6; 11.3%), and *Finegoldia magna* (*N* = 5; 9.4%). The only factor associated with wound improvement after a 1-month follow-up was the presence of *E. faecalis* (*p* = 0.012) when compared with patients without wound improvement. This study confirms the complementary role of culturomics in the exploration of complex microbiota. Further studies on a larger scale are needed to fully understand the clinical importance of the microbiota of diabetic foot infections.

## Introduction

Diabetes mellitus is considered to be one of the most widespread chronic diseases, with almost 10% of global adult population being diabetic or at risk of developing diabetes (World Health Organization, 2016). Nearly 15–20% of diabetic patients will suffer from a diabetic foot ulcer (DFU) during their lifetime (Singh et al., 2005). This morbidity is due to several factors combining poor glycaemia control, a peripheral neuropathy, peripheral vascular disease, poor hygiene habits, and trauma or micro-lesions of the foot (Lipsky et al., 2012). Osteomyelitis is a frequent complication of diabetic foot infections (DFI) and is related to more than 20% of moderate infections and 50–60% of severe infections (Lipsky et al., 2016). DFI is also a well-recognized risk factor for major amputation in diabetic patients (Lavery et al., 2006).

In contrast to DFU, DFI is defined by the presence of an inflammatory response and tissue injury that can run the clinical spectrum from superficial cellulitis to chronic osteomyelitis, being a consequence of interaction between the host and multiplying bacteria (Williams et al., 2004). Due to the confounding effect of neuropathy and ischemia on the local and systemic inflammatory response, diagnosing DFI in diabetic individuals is often difficult. As microorganisms are always present on skin wounds, DFUs have usually a polymicrobial nature, making the accuracy of bacterial identification challenging (Jneid et al., 2017). Recent studies on the microbiota of DFI suggested that molecular techniques, such as 16S rRNA PCR amplification, identify a greater diversity of bacteria and reveal more fastidious anaerobes and Gram-negative species than standard culture methods. However, molecular tools for bacterial identification are hampered by several biases (Lagier et al., 2015). Remarkably, molecular studies overlook minority species and cannot distinguish between living and dead bacteria. Moreover, they do not enable an assessment of antibiotic susceptibility.

A study under optimized anaerobic conditions reported that a wide range of anaerobes were cultured from DFIs (Claros et al., 2013). Culturomics, which has recently been developed in our laboratory, consists of the use of large-scale culture conditions with colony identification by matrix-assisted laser desorption ionization time-of-flight mass spectrometry (MALDI-TOF) or 16S rRNA PCR, and has revealed the potential to dramatically enlarge the spectra of bacteria identified by culture methods (Lagier et al., 2015).

Here, we describe the first systematic analysis of the microbiota of samples (*N* = 43) from patients with DFI using culturomics, in relation to clinical factors that may influence this microbiota. We report the bacterial composition, diversity and richness of the wound samples from patients with DFI and examine the clinical factors and treatment outcomes associated with DFI microbiota.

## Materials and methods

### Study design

This study was approved by the CHU Nimes institutional review board and carried out in accordance with the Helsinki Declaration, as revised in 2000. All patients gave written informed consent for their participation in the study. Between October 1, 2013 and December 31, 2013, all diabetic patients managed in the two diabetology departments of the Nîmes University Hospital who were suspected to have a new or aggravating episode of DFI, were included prospectively and consecutively in the study. The following criteria were used: over the age of 18, clinical signs of infection, and absence of osteomyelitis. All wounds were assessed for the presence and severity of infection (grade 2–4) by a trained diabetologist using the PEDIS (Perfusion, Extent, Depth, Infection, and Sensation) classification system (Lipsky et al., 2012). All patients had a systematic follow-up visit 1 month later. The following clinical data were recorded at the time of sampling by clinicians: demographic data, diagnosis at admission, PEDIS grade of wound severity, topography, number, ulcer size (including surface area and depth of the wound), diabetes type, duration of diabetes, HbA1C value, previous history of DFU, underlying diseases and their severity according to the Charlson score, and antibiotic therapy in the 14 previous days. When patients had several wounds, the sample was taken from the most severe wound. DFI management was performed according to international guidelines (Lipsky et al., 2012), independently of our study results. At the follow-up visit, the trained diabetologist noted the evolution of the most severe wound (PEDIS grade, size, and depth) and the antibiotics used.

### Samples

After wound debridement, samples for bacterial culture were obtained by scraping the wound base, collecting debris, and swabbing the wound base (the swab was rotated over a 1 cm^2^ area of the viable non-necrotic wound tissue). The samples were immediately placed into a transport medium (Eswab®, Becton Dickinson, New Jersey, USA). All samples were sent within an hour to the Department of Microbiology. They were then frozen at −80°C and were then sent to the Unité de Recherche Microbes Evolution Phylogenie et Infections (MEPHI) at Marseille to be cultured and analyzed. Transport tubes were degasified over the previous 3 days by incubating them with anaerobic generators (Anaerogen Thermo Scientific, MA USA).

### Culturomics method

Each sample (50 μL) was inoculated into aerobic and anaerobic blood-culture bottles enriched with 2.5 mL sheep blood and 5 mL rumen filtrated at 0.2 microns as previously described (Lagier et al., 2012). After incubation at 37°C for 24 h, 100 μL of the solution was drawn out of the blood-culture bottle and added to 900 μL of phosphate buffered saline (PBS). A 10-fold serial dilution was then performed, ranging from 10^−1^ to 10^−6^. All obtained dilutions were plated under seven preselected culture conditions (Table S1). Anaerobic incubation was performed in an anaerobic chamber (AES Chemunex, Combourg, France). Identification of the bacterial colonies was first performed by MALDI-TOF (Autoflex II®, Bruker Daltonik, Germany), as previously described (Seng et al., 2009). Isolates that were not identified by MALDI-TOF were submitted for molecular identification using 16S rRNA gene amplification and sequencing (Lagier et al., 2012).

### Statistical analysis

Descriptive statistics included percentages for the categorical variables and medians with interquartile ranges (IQR) for the non-normally distributed continuous variables. The comparisons were performed using the Chi-square test or Fisher's exact test for the categorical variables. The correlation analysis was performed by assessing the Spearman's rho coefficient of correlation. A *P-*value < 0.05 was considered to be statistically significant. Statistical analysis was performed using SPSS statistics 2016 (IBM, NY, USA). The principal component analysis was performed with XLSTAT 2017 (Addinsoft, Paris, France). Because richness (defined as the number of different species) does not take into account the abundance of species, we used the Shannon index to compare diversity (taking abundance into account), as previously described (Wang et al., 2008).

## Results

### Clinical data

In total, 43 patients were hospitalized for an episode of DFI and were enrolled into the study. The demographic and clinical characteristics of these patients are presented in Table S2.

Most of the included patients were male (69.7%) with a median age of 67.5 years (27–90). Thirty-eight patients (88.4%) had Type 2 diabetes. Twenty (46.5%) of the DFI were located on the plantar forefoot. Overall, the mean hemoglobin A1C was 7.16% (±1.13), the mean ulcer surface area was 1.7 cm (±1.9), and the mean ulcer depth was 1.22 cm (±1.36). Eighteen (58.1%) of the DFI were classified as grade 2 (PEDIS score), while the remainder were classified as above grade 2. For 31 patients (72%), the current wound was the first episode of DFI and 12 (27.9%) were included during a relapse of a previous DFU or DFI. Most of the selected patients (34, 79%) had not taken antibiotics in the previous 14 days. After a 1-month follow-up appointment and a standardized care in accordance with international guidelines, 25 (58.1%) of these patients had a favorable outcome with an evolution toward healing (Table S2).

### Culturomics results

**Figure 1:**
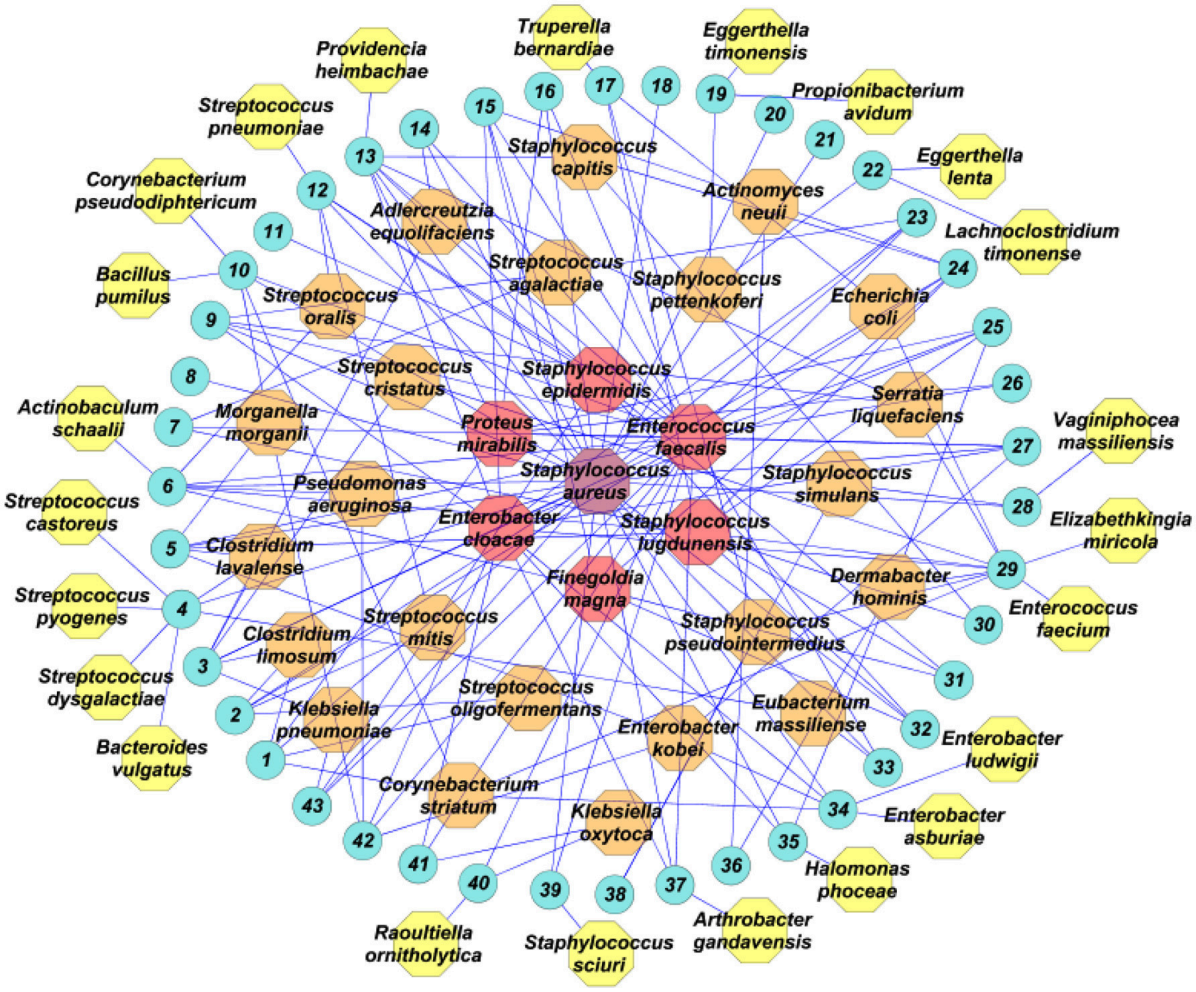
Bacterial species isolated from wound samples from 43 patients (blue circles), analyzed using culturomics. In purple, the most prevalent bacterial species; in red, the bacterial species isolated in five or more patients; in orange, the bacterial species isolated in more than one patient; in yellow, species isolated in only one patient.

We analyzed DFI microbiota using culturomics. We generated ~30,000 colonies with an average of 698 colonies per sample. A total of 53 different bacterial species were identified (Table S3). The majority were classified as belonging to four phyla: *Actinobacteria* (15.09%)*, Bacteroidetes* (3.77%)*, Firmicutes* (52.83%), and *Proteobacteria* (26.41%). To compare bacterial diversity among the infecting ulcers, the global α-Shannon index was determined, an ecological measure of diversity that contains the total number of different species and their relative proportions. The higher the Shannon index values, the greater the diversity. The α-Shannon index was 3.34 when considering all samples, suggesting great heterogeneity in the DFI. Of all the identified bacterial species, 30 (56.6%) were shared by at least two samples and 23 (43.4%) were isolated only once (Figure 1). The majority (44; 81.4%) of species had previously been cultured from human stool samples as part of the human gut microbiota study, and 19 (35.8%) had never been cultured or identified by molecular methods from a DFU (Table S3). Five species (9.4%) (*Raoultella ornithinolytica, Eubacterium massiliense, Eggerthella timonensis, Lachnoclostridium timonense, and Vaginiphocea massiliensis*) had previously been described as new species by our laboratory as part of the Culturomics Project (Lagier et al., 2016). Bacterial richness, defined by the median number and standard deviation of different species isolated per patient, was 4 ± 2 in this study. Most samples were polymicrobial (38, 88.3%) corresponding to a mean number of 4.07 isolates per sample.

Analysis of the culturomics results showed that aerobic Gram-positive bacteria were the most frequent species isolated (29, 54.7%), followed by aerobic Gram-negative bacteria (14, 26.4%) and strict anaerobes (10, 18.9%). At the patient level, aerobic Gram-positive were isolated in all patients (43, 100%), followed by aerobic Gram-negative bacteria (26, 60.5%) and strict anaerobes (14, 32.6%). The most prevalent Gram-positive species were *Staphylococcus aureus* (28, 52.8%), *Enterococcus faecalis* (24, 45.2%), *Staphylococcus lugdunensis* (10, 18.7%), and *Staphylococcus epidermidis* (6, 11.3%). The most prevalent Gram-negative bacteria were *Enterobacter cloacae* (12, 22.6%) and *Proteus mirabilis* (6, 11.3%). The most prevalent strict anaerobe was *Finegoldia magna* (5, 9.4%; Figure 1).

### Influence of DFI microbiota and evolution of the wound

**Figure 2:**
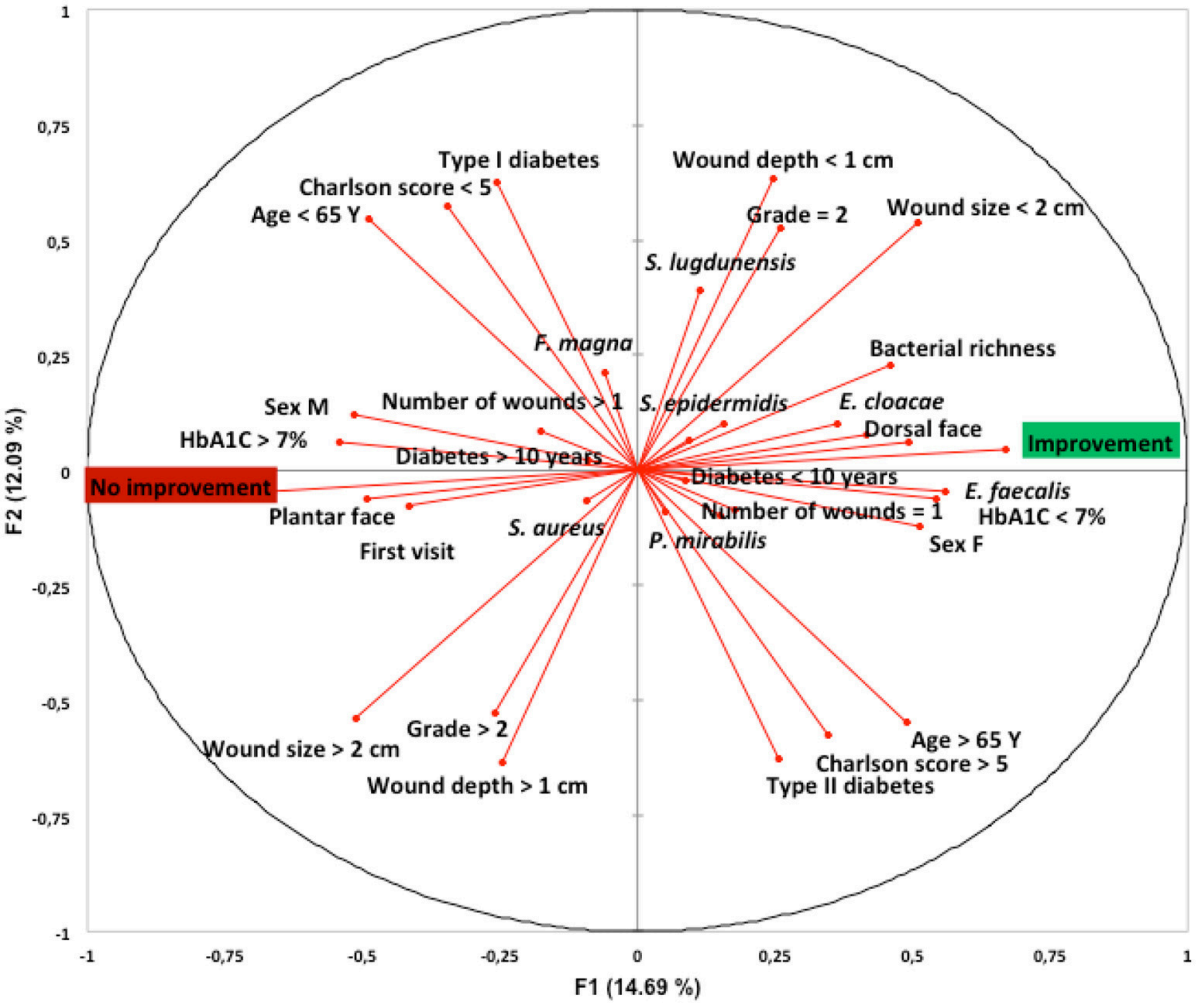
Link between patients and wound characteristics, bacterial composition and clinical outcome at 1-month follow-up appointment and following standardized treatment. Principal component analysis using XLSTAT-2017 (Addinsoft, Paris, France) was performed on species' raw data (presence on each patient's wound sample) obtained by culturomics. The first (F1), and second (F2) components accounted for 17.5 and 11.7%, respectively, of the overall variability.Y, years; F, female; M, male; DFU, diabetic foot ulcer; *S. aureus, Staphylococcus aureus*; *S. lugdunensis, Staphylococcus lugdunensis*; *S. epidermidid, Staphylococcus epidermidis*; *E. faecalis, Enterococcus faecalis*; *E. cloacae, Enterobacter cloacae*; *F. magna, Finegoldia magna*; *P. mirabilis, Proteus mirabilis*.

We determined whether clinical factors and the most frequently isolated bacterial species of the DFI microbiota were associated with evolution of the ulcer. Principal component analysis suggested that the factors associated with wound improvement at the 1-month follow-up appointment were HbA1C <7%, age >65 years, female, DFI location in the dorsal face, bacterial richness (number of different species isolated per wound sample), Type 2 diabetes, duration of diabetes <10 years, a Charlson score >5, a PEDIS grade of 2, a wound size <2 cm, a wound depth <1 cm, the presence of only one wound, and the presence in the wound sample of *S. lugdunensis, S. epidermidis, E. cloacae, P. mirabilis*, and *E. faecalis*. Conversely, principal component analysis suggested that the factor associated with unfavorable wound evolution at the 1-month follow-up appointment were HbA1C >7%, age <65 years, male, DFI location in the plantar face, Type 1 diabetes, duration of diabetes >10 years, a Charlson score <5, a PEDIS grade above 2, a wound size >2 cm, a wound depth >1 cm, the presence of more than one wound, the presence in the wound sample of *S. aureus* and *F. magna* (Figure 2).

Further investigation by univariate analysis showed that the presence of *E. faecalis* was significantly associated with wound improvement at the 1-month follow-up appointment, when compared to patients without wound improvement (*p* = 0.012; Table 1). The only two factors significantly associated with unfavorable wound evolution at the 1-month follow-up appointment was a PEDIS grade above 2 (*p* = 0.005), and the length of diabetes (*p* = 0.015).

**Table 1:** Demographic and clinical characteristics of 43 patients with DFI together with the presence of bacterial species in their wound samples were analyzed.

**Characteristics**	**Value**			***P***
	**Improvement** ***n* = 25**	**No improvement** ***n* = 18**	**Total** ***n* = 43**	**Improvement vs. no** ** improvement**
Age (mean ± SD), y	67.84 (15.96)	64.66 (13.87)	66.51 (15.03)	0.557
Male/female, *n* (%)	17 (68)/8 (32)	14 (77.77)/4 (22.22)	31 (72.09)/12 (27.9)	0.513
Type 1/Type 2 diabetes mellitus, *n* (%)	2 (8)/23 (92)	3 (17) / 15 (83)	5 (12)/38 (88)	0.681
Clinical context (first visit/ follow up), *n* (%)	17 (68)/8 (32)	13 (72)/5 (28)	30 (70)/13 (30)	0.785
PEDIS severity grade, *n* (%)				
2 3 4	15 (60) 10 (40) 0 (0)	3 (17) 14 (78) 1 (5)	18 (42) 24 (56) 1 (2)	0.005^*^
Charlson score> 5, *n* (%)	12 (48)	2 (11)	14 (33)	0.113
Diabetes duration, (mean ± SD), y	17.04 (7.02)	20.25 (12.1)	18.38 (9.48)	0.015^*^
HbA1C, (mean ± SD), %	7.016 (0.94)	7.35 (1.35)	7.15 (1.12)	0.421
Previous antibiotics, *n* (%)	7 (28)/18 (72)	3 (17)/15 (83)	10 (23)/33 (77)	0.620
Number of wounds, *n* (%)				
1 2 3 4	20 (80) 3 (12) 1 (4) 1 (4)	14 (78) 3 (17) 0 (0) 1 (5)	34 (79) 6 (14) 1 (2) 2 (5)	0.862
Wound size, (mean ± SD), mm	12.75 (16)	20.37 (17)	15.8 (16)	0.769
Wound depth, (mean ± SD), mm	15.3 (13)	18.45 (10)	16.65 (12)	0.268
Plantar/dorsal face	8 (32)/17 (68)	9 (50)/9 (50)	18 (42)/26 (58)	0.254
*Staphylococcus aureus, n* (%)	17 (68)	11 (61)	28 (65)	0.862
*Enterobacter cloacae, n* (%)	7 (28)	5 (28)	12 (28)	0.163
*Proteus mirabilis, n* (%)	4 (16)	2 (11)	6 (14)	0.648
*Enterococcus faecalis, n* (%)	17 (68)	7 (39)	24 (56)	0.012^*^
*Staphylococcus lugdunensis, n* (%)	6 (24)	4 (22)	10 (23)	0.891
*Staphylococcus epidermidis, n* (%)	3 (12)	2 (11)	5 (12)	0.757
Strict anaerobes	7 (28)	10 (55)	17 (40)	0.068
*Finegoldia magna, n* (%)	2 (8)	3 (17)	5 (12)	0.382

Factors associated, by univariate analysis, with wound improvement at the 1-month follow-up appointment and following standardized treatment. DFI, diabetic foot infection; SD, standard deviation; y, years.

* *Statistically significant*.

We found a correlation between the presence of *E. cloacae* with *E. faecalis* (rho = 0.523; *p* = 0.026) and *S. lugdunensis* with *F. magna* (rho = 0.478; *p* = 0.045) in wound samples from patients who had an unfavorable outcome at the 1-month follow-up appointment, and between the presence of *S. aureus* with *S. lugdunensis* (rho = 0.418; *p* = 0.018), *E. cloacae* with *P. mirabilis* (rho = 0.457; *p* = 0.022), and *S. lugdunensis* with *F. magna* (rho = 0.478; *p* = 0.045) in patients who had a wound improvement at their 1-month follow-up appointment (Table S4).

## Discussion

This study described for the first time, to our knowledge, the DFI microbiota using culturomics methods. With this technology, we observed a high bacterial diversity (number of different bacteria isolated = 53) which represents the highest number of different bacterial species cultured from DFI in a single study (Jneid et al., 2017). Of particular note is the fact that 35.8% of the identified bacteria had not been isolated from DFI in previous studies (Jneid et al., 2017). Indeed, while metagenomics provides a fairly good analysis of the presence of majority flora, particularly in terms of not yet cultivable bacteria, culturomics is likely to provide a better assessment of the diversity of minority flora, especially for relatively poor flora such as in DFI. The role of minoritary species on the evolution or the prognosis of the disease should be further investigated to evaluate its value for clinicians but culturomics and bacterial metagenomics appear to be two complementary tools for studying the human microbiota (Jneid et al., 2017).

The first key observation of this study is that most of the samples were polymicrobial (88.3%). While this has traditionally been the case of DFUs, most of the studies presented bacterial ecology without debridement, including commensal and pathogenic bacteria. However, even when debridement was performed, it remained difficult to assess the role of these bacteria and, in particular, to distinguish pathogenic bacteria from commensal ones. The most recent guidelines on managing DFI are based on standard culture-dependent bacterial identification and the administration of a narrow spectrum of antibiotics based on sensitivity results (Lipsky et al., 2016). However, success rates are far from satisfactory, with rarely more than half of the patients responding positively to this approach (Pereira et al., 2017). Recently, Dowd et al. introduced the concept of the “functionally equivalent pathogroup,” responsible for chronicity of the infection and maintenance of the pathogenic biofilm (Dowd et al., 2008). Moreover, in a prospective, randomized, multi-center trial comparing antibiotic regimens for the treatment of DFI, MRSA-positive patients exhibited positive responses to non-anti-MRSA antibiotic treatments (Lipsky et al., 2005). Furthermore, in a recent study by Malone et al. the authors used next-generation DNA sequencing to analyze the DFI microbiota and found no overall differences in the microbiomes of patients whose treatment failed and those who experienced treatment success with directed antimicrobial therapy based on conventional bacterial cultures (Malone et al., 2017). Therefore, it is still unclear whether assessing the bacterial diversity of DFI samples may have an impact on clinical practice.

The second observation is that *S. aureus* was the most prevalent (52.8%) bacteria isolated in the different samples, and the presence of *S. aureus* in the DFI was associated with a worsening of the wound (Figure 2). This result is consistent with previous studies, although the bacterial diversity and prevalence of specific bacteria vary greatly between studies (Dunyach-Remy et al., 2016). Indeed, the diabetic foot microbiota is influenced by several factors such as demographic characteristics, personal hygiene, grade of severity, glycemic control and ongoing or previous antibiotic treatments, as well as by the bacterial identification method used (Jneid et al., 2017). However, the geographical origin of the patient seems to be one of the most important factors. Indeed, in warmer countries (particularly in Asia and Africa), Gram-negative bacilli are more prevalent compared to western countries (Dunyach-Remy et al., 2016). Two large culture-dependent studies in western countries on the ecology of DFIs which sampled over 1,266 patients revealed similar results (Ge et al., 2002; Citron et al., 2007). They identified mostly Gram-positive aerobic bacteria, primarily *Staphylococcus* spp. (24–35%) and especially *S. aureus* (47–55%). A higher incidence of Gram-negative aerobes (*P. aeruginosa*, Enterobacteriaceae) and anaerobes was found in the most chronic wounds (Citron et al., 2007). Gardner et al. profiled the microbiome of 52 individuals with non-infected DFUs using 16S rDNA pyrosequencing (Gardner et al., 2013). *Staphylococcus* spp. was present in 49 of 52 DFU samples, comprising 29.6% of the total sequences. The majority of *Staphylococcus* spp. sequences (96.5%) were classified as *S. aureus*. Interestingly, culturomics analysis showed that anaerobes represent 32.6% of the DFI samples. A recent meta-analysis noted that the unweighted average of anaerobes identified by molecular techniques was 11% of all isolates (Charles et al., 2015). The true frequency of anaerobes in DFI remains unclear, largely related to a large variety of bacterial culture methods, the types of sample taken for analysis, and the transport media used. Anaerobes are predominantly seen in DFI with ulcers that are deeper, and more chronic, associated with ischemia, necrosis, gangrene, or a foul odor (Charles et al., 2015). In line with these results, through a principal component analysis of our culturomics approach, we observed that the presence of *F. magna* was associated with an unfavorable outcome in relation to ulcers at 1 month and, although not statistically significant, we identified strict anaerobic species in more patients with no improvement. However, the majority of the wounds analyzed in this study were superficial (PEDIS grade 2). Therefore, the prevalence of anaerobes reported here (32%) probably falls well below that of deeper wounds.

Univariate analysis, suggested that the main factor associated significantly with wound improvement at the 1-month follow-up appointment and following standardized treatment was the presence of *E. faecalis*. Although this result needs further confirmation on a larger scale, it is relevant since some *E. faecalis* strains have been described as probiotics (Franz et al., 2011). Moreover, a study by Lipsky et al. noted that the clinical response rates were similar for ertapenem and piperacillin/tazobactam in patients with isolates of *Enterococcus* spp. (86.8 vs. 80.8%, respectively), despite the fact that these isolates were resistant to the former but not to the latter agent (Lipsky et al., 2005), suggesting the low virulence potential of this species. Interestingly, using principal component analysis, we found an association between the presence in the wound of some coagulase-negative staphylococci (*S. epidermidis, S. lugdunensis*) and wound improvement. This is consistent with recent studies by Zipperer et al. who observed that human nasal colonization by *S. lugdunensis*, which produces lugdunin, was associated with a significantly reduced *S. aureus* carriage rate (Zipperer et al., 2016). In a previous study, our team also showed that commensal Gram-positive cocci, *Helcococcus kunzii*, frequently associated with *S. aureus* in DFI, decreased the virulence of this pathogenic bacterium. These results suggest the presence of an important network of cell-to-cell communication between commensal and pathogenic bacteria in DFI, with this being valuable for preventing staphylococcal infections.

Our study has several limitations that warrant further study. First, due to the small sample size (*N* = 43), any clinical inferences between patients, bacteria and clinical outcome need to be validated in a larger study. Furthermore, the study is monocentric. However, the number of patients represents an significant volume for a pilot study and patients were recruited from the national reference center for DFI, which specializes in the management of samples (Zipperer et al., 2016). Second, antimicrobial susceptibility testing was not performed on our bacterial isolates, basically because of the high number of colonies obtained by culturomics (*N* = 30,000). It should be noted, however, that studies using new molecular techniques have the same limitations (Lavigne et al., 2015). Finally a last limitation of this study is the number of bacterial species likely missed by culturomics. Despite bacterial metagenomics presents several bias, simultaneous analysis would better appreciate bacterial species missed by culturomics and proportion of different bacterial species. Indeed, the need for enrichment broth in the culturomics procedure does not allow to determine the real proportion of the different bacterial species in the wound. Using cryopreservative before freezing samples such as recently engineered swabs with amies buffer containing glycerol (http://www.copanusa.com/files/2114/7224/0314/ASM_2016_ESwab_20_glycerol_poster.pdf) or inoculation of culture broth at the bedside would also probably increase the number fastidious bacteria identified.

## Conclusions

This study provides a useful insight into the bacterial composition of samples from DFI. It confirms the complementary role of culturomics in relation to molecular methods in the exploration of complex microbiota. Although this new knowledge on the bacterial composition of wounds has not yet led to useful improvements in clinical practice, an exciting area of research would be microbiota transplants in the skin (or wounds), as has already been tested for atopic dermatitis (Nakatsuji et al., 2017).

## Author contributions

JJ performed culture isolation and identification. SS, NJ, AS, and J-PL received patients in consultations and they collected samples. J-PL and BL conceived the study. JJ, NC, J-PL, and BL analyzed the results and wrote the manuscript. All authors approved the final version.

### Conflict of interest statement

The authors declare that the research was conducted in the absence of any commercial or financial relationships that could be construed as a potential conflict of interest.
