# Supplementary material for: Exploring the Microbiota of Diabetic Foot Infections With Culturomics
Source: Front Cell Infect Microbiol. 2018 Aug 14;8:282. doi: 10.3389/fcimb.2018.00282 (PMC6102383; doi:10.3389/fcimb.2018.00282)
Supplement: Supplementary file 1 [file Data_Sheet_1.PDF]

Table S1: Culture media and conditions used for the culturomics study

Culture condition	Conditions	Provenance
5% Columbia sheep blood agar, 37°C	Aerobe, Anaerobe	(Biomérieux)
BHI (Brain Heart Infusion) supplemented with kanamycine and vancomycine, 37°C	Aerobe, Anaerobe	(Biomérieux)
Agar medium supplemented with blood sheep and ascorbic acid, 37°C	Aerobe	(Biomérieux)
ANC (acide nalidixique colistine)	Aerobe	(Biomérieux)
BCP (Bromocrésol pourpre)	Aerobe	(Biomérieux)

Table S2: Clinical characteristics of 43 patients with diabetic foot infection analysed in this study

Patient	Sex/Age (years)	Clinical context	PEDIS severity grade	CHARLSON score	Diabetes type/duration	HbA1C (%)	ATB in last 14 days	Number of wounds	Wound size (mm)	Wound depth (mm)	Localization	Evolution
1	M / 59	1st visit	2	3	Type II / 11 years	6.1	No	1	15 x 10	2	Right foot: Plantar face	No improvement
2	M / 90	Follow up	2	10	Type II / 16 years	6.3	No	2	12 x 10	N.A.	Foot: Toe	Wound improving
3	M / 85	1st visit	2	6	Type II / 22 years	6.7	No	1	7 x 3	N.A.	Right foot: Toe	Wound improving
4	M / 38	1st visit	3	3	Type I / 20 years	6	No	2	15 x 3	N.A.	Left foot: Plantar face; Heel	No improvement
5	M / 63	Follow up	2	10	Type II / 21 years	8.1	Yes	1	6 x 4	2	Right foot	Wound improving
6	M / 66	1st visit	2	4	Type II / 14 years	6.9	No	1	4 x 2	1	Left foot: Dorsal face	Wound improving
7	M / 66	1st visit	2	5	Type II / 16 years	6.9	No	1	5 x 4	10	Left foot: Dorsal face; Toe	Wound improving
8	M / 76	1st visit	3	9	Type II / 24 years	7.2	No	1	60 x 40	N.A.	Foot: Toe	Wound improving
9	F / 87	1st visit	3	5	Type II / 22 years	6.8	Yes	1	12 x 9	20	Right foot: Dorsal face; Toe	No improvement
10	F / 81	1st visit	3	7	Type II / 11 years	6.8	No	1	45 x 24	30	Right leg: Under malleolus; Interior side	Wound improving

11	F / 66	1st visit	3	5	Type II / 13 years	7.6	No	1	8 x 2	N.A.	Right foot: Dorsal face; Toe	No improvement
12	M / 66	1st visit	2	8	Type II / 6 years	6.5	No	1	3 x 2	N.A.	Right foot: Dorsal face; Toe	Wound improving
13	F / 70	1st visit	2	6	Type II / 12 years	6.5	Yes	1	12 x 6	10	Right foot: Plantar face	Wound improving
14	M / 27	1st visit	2	5	Type I / 13 years	7.5	Yes	1	5 x 5	N.A.	Right foot: Dorsal face, toe	Wound improving
15	F / 51	1st visit	3	4	Type II / 35 years	8.2	No	1	N.A.	N.A.	Foot: Plantar face	No improvement
16	F / 73	1st visit	3	5	Type II / 12 years	6.7	No	1	15 x 12	30	Right foot: Dorsal face; Metatarsus	Wound improving
17	M / 66	1st visit	3	4	Type II / 18 years	7.2	No	1	5 x 5	N.A.	Foot: Dorsal face; Toe	Toe amputation
18	F / 46	1st visit	2	4	Type I / 13 years	6.3	No	4	9 x 8	N.A.	Foot: Plantar face; Toe	Wound improving
19	M / 56	1st visit	3	5	Type II / 25 years	7.2	No	1	30 x 30	20	Left foot: Plantar face; Metatarsus	No improvement
20	M / 75	1st visit	3	3	Type II / 11 years	7.3	No	1	4 x 4	30	Right foot: Dorsal face; Toe	Wound improving
21	M / 80	Follow up	2	4	Type II / 10 years	7.5	No	1	30 x 25	N.A.	Left foot: Plantar face, Metatarsus	No improvement
22	M / 50	Follow up	2	5	Type II / 20 years	6.1	No	1	40 x 30	5	Right foot: Plantar face	Wound improving
23	F / 78	1st visit	3	5	Type II / 15	6.4	Yes	1	14 x 9	30	Left foot:	No

					years						Dorsal face	improvement
24	M / 70	Follow up	3	5	Type II / 49 years	6.5	No	2	15 x 18	25	Foot: Dorsal face; Metatarsus	No improvement
25	M / 66	1st visit	2	4	Type II / 14 years	6.9	No	1	4 x 2	1	Left foot: Dorsal face	Wound improving
26	M / 70	1st visit	3	7	Type II / 17 years	6.9	No	1	22 x 16	30	Right foot: Dorsal face; Toe	No improvement – Toe amputation
27	M / 64	1st visit	2	9	Type II / 26 years	9	No	1	20 x 15	N.A.	Foot : Plantar face and right heel	Wound improving
28	M / 81	1st visit	4	1	Type II / 23 years	7.3	No	1	22 x 15	30	Right foot: Plantar face; Metatarsus	No improvement
29	F / 90	Follow up	2	5	Type II / 11 years	6.9	Yes	1	6 x 4	8	Left foot: Dorsal face; Toe	Wound improving
30	M / 42	1st visit	2	3	Type I / 17 years	7.4	No	1	7 x 6	15	Left foot: Dorsal face; Toe	No improvement
31	M / 34	1st visit	2	7	Type II / 12 years	7.5	No	1	10 x 7	10	Left foot: Dorsal face; Toe	Wound improving
32	M / 56	Follow up	3	3	Type II / 18 years	6.3	No	1	2 x 2	20	Foot: Plantar face, Toe	No improvement
33	M / 72	Follow up	3	5	Type II / 21 years	6	Yes	1	5 x 5	42	Right foot: Plantar face; Toe	Wound improving
34	M / 57	1st visit	3	3	Type II / 14 years	7.2	No	2	35 x 30	N.A.	Foot: Plantar face, toe	No improvement

35	M / 83	Follow up	3	6	Type II / 29 years	8.8	No	1	5 x 5	10	Left foot: Dorsal face, Metatarsus	Wound improving
36	M / 83	1st visit	3	5	Type II / 12 years	6.7	No	1	74 x 60	5	Left foot: Plantar face; Heel	No improvement
37	M / 69	Follow up	3	5	Type II / 25 years	5	No	3	N.A.	N.A.	Right foot: Dorsal face; Metatarsus	Wound improving
38	F / 78	1st visit	3	6	Type II / 36 years	6.9	Yes	2	10 x 10	25	Foot: Plantar face, Metatarsus	Wound improving
39	F / 60	Follow up	3	6	Type II / 12 years	7.3	Yes	1	4 x 3	N.A.	Foot: Dorsal face	Wound improving
40	M / 59	1st visit	3	5	Type II / 18 years	9	No	2	12 x 12	16	Left foot: Metatarsus	Wound improving
41	M / 58	Follow up	3	4	Type I / 45 years	9.2	Yes	1	N.A.	N.A.	Foot: Dorsal face, toe	No improvement
42	F / 87	1st visit	2	3	Type II / 11 years	6.3	No	1	3 x 3	N.A.	Left foot: Dorsal face, toe	Wound improving
43	M / 66	Follow up	3	7	Type II / 6 months	11.8	No	4	20 x 15	6	Foot: External side	No improvement

Table S3: Bacterial species (N=53) isolated from DFI (N=43) and their characteristics

Bacterial species	Isolates	Aerotolerance	Phylotype	Human microbiota	DFU
Staphylococcus aureus	28	Aerobic/anaerobic	Firmicutes	(Lagier et al., 2016)	(Jneid et al.)
Enterococcus faecalis	24	Aerobic/anaerobic	Firmicutes	(Lagier et al., 2016)	(Jneid et al.)
Enterobacter cloacae	12	Aerobic/anaerobic	Proteobacteriae	(Lagier et al., 2016)	(Jneid et al.)
Staphylococcus lugdunensis	10	Aerobic/anaerobic	Firmicutes	(Lagier et al., 2016)	(Jneid et al.)
Staphylococcus epidermidis	6	Aerobic/anaerobic	Firmicutes	(Lagier et al., 2016)	(Jneid et al.)
Proteus mirabilis	6	Aerobic/anaerobic	Proteobacteriae	(Lagier et al., 2016)	(Jneid et al.)
Finegoldia magna	5	Anaerobic	Firmicutes	(Lagier et al., 2016)	(Jneid et al.)[2](2)[2]
Enterobacter kobei	4	Aerobic/anaerobic	Proteobacteriae	(Lagier et al., 2016)	-
Streptococcus oralis/mitis	4	Aerobic/anaerobic	Firmicutes	(Lagier et al., 2016)	(Citron et al., 2007) (Jneid et al.)
Dermabacter hominis	4	Aerobic/anaerobic	Actinobacteriae	(Lagier et al., 2016)	(Jneid et al.)
Corynebacterium striatum	3	Aerobic/anaerobic	Actinobacteriae	(Lagier et al., 2016)	(Jneid et al.)
Morganella morganii	3	Aerobic/anaerobic	Proteobacteriae	(Lagier et al., 2016)	(Jneid et al.)
Serratia liquefaciens	3	Aerobic/anaerobic	Proteobacteriae	(Grohskopf et al., 2001)	(Jneid et al.)
Streptococcus agalactiae	3	Aerobic/anaerobic	Firmicutes	(Lagier et al., 2016)	(Jneid et al.)
Staphylococcus capitis	3	Aerobic/anaerobic	Firmicutes	(Lagier et al., 2016)	(Galkowska et al., 2009)
Actinomyces neuii	3	Aerobic/anaerobic	Actinobacteriae	(Lagier et al., 2016)	(Jneid et al.)
Clostridium limosum	3	Anaerobic	Firmicutes	(Just et al., 1992)	-
Clostridium lavalense	3	Anaerobic	Firmicutes	(Lagier et al., 2016)	-
Staphylococcus pseudointermedius	3	Aerobic/anaerobic	Firmicutes	(Van Hoovels et al., 2006)	-
Pseudomonas aeruginosa	2	Aerobic/anaerobic	Proteobacteriae	(Lagier et al., 2016)	(Jneid et al.)
Klebsiella pneumoniae	2	Aerobic/anaerobic	Proteobacteriae	(Lagier et al., 2016)	(Jneid et al.)

Klebsiella oxytoca	2	Aerobic/anaerobic	Proteobacteriae	(Lagier et al., 2016)	(Jneid et al.)
Escherichia coli	2	Aerobic/anaerobic	Proteobacteriae	(Lagier et al., 2016)	(Jneid et al.)
Streptococcus oligofermentans	2	Aerobic/anaerobic	Firmicutes	(Tong et al., 2003)	-
Adlercreutzia equolifaciens	2	Anaerobic	Firmicutes	(Lagier et al., 2016)	-
Streptococcus cristatus	2	Aerobic/anaerobic	Firmicutes	(Lagier et al., 2016)	-
Staphylococcus pettenkoferi	2	Aerobic/anaerobic	Firmicutes	(Lagier et al., 2016)	(Jneid et al.)
Staphylococcus simulans	2	Aerobic/anaerobic	Firmicutes	(Lagier et al., 2016)	(Jneid et al.)
Eubacterium massiliense	2	culturomics	Firmicutes	(Lagier et al., 2016)	-
Streptococcus pyogenes	1	Aerobic/anaerobic	Firmicutes	(Talay et al., 1992)	(Stappers et al., 2015)
Providencia heimbachae	1	Aerobic/anaerobic	Proteobacteriae	(Lagier et al., 2016)	(Jneid et al., 2016)
Elizabethkingia miricola	1	Aerobic	Bacteroidetes	(Lagier et al., 2016)	(Pentima, 2010)
Enterobacter asburiae	1	Aerobic/anaerobic	Proteobacteriae	(Lagier et al., 2016)	-
Enterobacter ludwigii	1	Aerobic/anaerobic	Proteobacteriae	(Lagier et al., 2016)	-
Halomonas phoceae	1	Aerobic/anaerobic; halophilic	Proteobacteriae	(Berger et al.)	-
Raoultella ornithinolytica	1	Aerobic	Proteobacteriae	(Lagier et al., 2016)	(Solak et al., 2011)
Actinobaculum schaalii	1	Aerobic/anaerobic	Actinobacteriae	(Fendukly and Osterman, 2005)	(Jneid et al.)
Bacillus pumilus	1	Aerobic	Firmicutes	(Lagier et al., 2016)	-
Corynebacterium pseudodiphtericum	1	Aerobic/anaerobic	Actinobacteriae	(Lagier et al., 2016)	-
Streptococcus pneumoniae	1	Aerobic/anaerobic	Firmicutes	(Lagier et al., 2016)	(Jneid et al.)
Enteroccoccus faecium	1	Aerobic/anaerobic	Firmicutes	(Lagier et al., 2016)	-
Streptococcus dysgalactiae	1	Aerobic/anaerobic	Firmicutes	(Lagier et al., 2016)	(Jneid et al.)
Streptococcus castoreus	1	Aerobic/anaerobic	Firmicutes	(Lagier et al., 2016)	-
Staphylococcus sciuri	1	Aerobic/anaerobic	Firmicutes	(Lagier et al., 2016)	(Marsou et al., 1999)
Arthrobacter	1	Aerobic/anaerobic	Firmicutes	(Lagier et	-

gandavensis				al., 2016)	
Bacteroides vulgatus	1	Anaerobic	Bacteroidetes	(Lagier et al., 2016)	(Jneid et al.)
Propionibacterium avidum	1	Anaerobic	Actinobacteriae	(Lagier et al., 2016)	(Jneid et al.)
Eggerthella timonensis	1	Anaerobic	Actinobacteriae	(Lagier et al., 2016)	–
Lachnoclostridium timonense	1	Anaerobic	Firmicutes	(Lagier et al., 2016)	–
Eggerthella lenta	1	Anaerobic	Actinobacteriae	(Lagier et al., 2016)	(Loïez et al., 2007)
Peptoniphilus olsenii	1	Anaerobic	Firmicutes	(Song et al., 2007)	(Loïez et al., 2007)
Vaginella massiliensis	1	culturomics	Firmicutes	(Lagier et al., 2016)	-

Tables S4: **Correlation table between each isolated bacterial species from patients who had an unfavourable outcome at 1-month follow-up (a), and patients who had a wound improvement at 1-month follow-up (b)**

Spearman's correlation coefficient rho was assessed. The analysis was performed by using SPSS statistics 2016 (IBM, NY, USA).

* Correlation significant at .05 level

** Correlation significant at .01 level

A

		S. aureus	*E. cloacae*	*P. mirabilis*	*E. faecalis*	*S. lugdunensis*	*F. magna*	*S. epidermidis*
S. aureus	rho	1.000	0.240	-0.081	0.169	-0.122	0.357	-0.081
	p	.	0.337	0.751	0.503	0.630	0.146	0.751
E. cloacae	rho	-	1.000	-0.219	0.523*	-0.331	0.055	-0.219
	p	-	.	0.382	0.026	0.179	0.827	0.382
P. mirabilis	rho	-	-	1.000	0.081	-0.189	-0.158	-0.125
	p	-	-	.	0.751	0.453	0.531	0.621
E. fecalis	rho	-	-	-	1.000	-0.152	0.255	-0.282
	p	-	-	-	.	0.546	0.307	0.257
S. lugdunensis	rho	-	-	-	-	1.000	0.478*	0.236
	p	-	-	-	-	.	0.045	0.345
F. magna	rho	-	-	-	-	-	1.000	0.316
	p	-	-	-	-	-	.	0.201
S. epidermidis	rho	-	-	-	-	-	-	1.000
	p	-	-	-	-	-	-	.

b

		S. aureus	*E. cloacae*	*P. mirabilis*	*E. faecalis*	*S. lugdunensis*	*F. magna*	*S. epidermidis*
S. aureus	rho	1.000	-0.145	0.065	0.265	-0.418*	-0.114	-0.011
	p	.	0.489	0.756	0.201	0.038	0.588	0.960
E. cloacae	rho	-	1.000	0.457*	0.046	0.067	-0.184	0.044
	p	-	.	0.022	0.828	0.751	0.379	0.835
P. mirabilis	rho	-	-	1.000	0.299	0.010	-0.129	0.175
	p	-	-	.	0.146	0.961	0.540	0.404
E. fecalis	rho	-	-	-	1.000	-0.016	-0.114	-0.011
	p	-	-	-	.	0.939	0.588	0.960
S. lugdunensis	rho	-	-	-	-	1.000	0.525**	0.081
	p	-	-	-	-	.	0.007	0.701
F. magna	rho	-	-	-	-	-	1.000	0.345
	p	-	-	-	-	-	.	0.091
S. epidermidis	rho	-	-	-	-	-	-	1.000
	p	-	-	-	-	-	-	.

References :

Berger, P., Adekambi, T., Raoult, D., and Drancourt, M. Halomonas phoceae sp. nov. isolated from human samples. NCBI 2005. *https://www.ncbi.nlm.nih.gov/Taxonomy/Browser/wwwtax.cgi?id=326994.*

Citron, D. M., Goldstein, E. J. C., Merriam, C. V., Lipsky, B. A., and Abramson, M. A. (2007). Bacteriology of moderate-to-severe diabetic foot infections and in vitro activity of antimicrobial agents. *J. Clin. Microbiol.* 45, 2819–2828. doi:10.1128/JCM.00551-07.

Fendukly, F., and Osterman, B. (2005). Isolation of Actinobaculum schaalii and Actinobaculum urinale from a patient with chronic renal failure. *J. Clin. Microbiol.* 43, 3567–3569. doi:10.1128/JCM.43.7.3567-3569.2005.

Galkowska, H., Podbielska, A., Olszewski, W. L., Stelmach, E., Luczak, M., Rosinski, G., et al. (2009). Epidemiology and prevalence of methicillin-resistant Staphylococcus aureus and Staphylococcus epidermidis in patients with diabetic foot ulcers: focus on the differences between species isolated from individuals with ischemic vs. neuropathic foot ulcers. *Diabetes Res. Clin. Pract.* 84, 187–193. doi:10.1016/j.diabres.2009.02.008.

Grohskopf, L. A., Roth, V. R., Feikin, D. R., Arduino, M. J., Carson, L. A., Tokars, J. I., et al. (2001). Serratia liquefaciens bloodstream infections from contamination of epoetin alfa at a hemodialysis center. *N. Engl. J. Med.* 344, 1491–1497. doi:10.1056/NEJM200105173442001.

Jneid, J., Benamar, S., Pagnier, I., Levy, P.-Y., Lavigne, J.-P., and La Scola, B. (2016). Draft Genome Sequence of Providencia heimbachae, Isolated from a Diabetic Foot Ulcer. *Genome Announc.* 4. doi:10.1128/genomeA.00276-16.

Jneid, J., Lavigne, J. P., La Scola, B., and Cassir, N. The diabetic foot microbiota: A review. *Hum. Microbiome J. 20175-6 1-6 Doiorg101016jhumic201709002*.

Just, I., Mohr, C., Schallehn, G., Menard, L., Didsbury, J. R., Vandekerckhove, J., et al. (1992). Purification and characterization of an ADP-ribosyltransferase produced by Clostridium limosum. *J. Biol. Chem.* 267, 10274–10280.

Lagier, J.-C., Khelaifia, S., Alou, M. T., Ndongo, S., Dione, N., Hugon, P., et al. (2016). Culture of previously uncultured members of the human gut microbiota by culturomics. *Nat. Microbiol.* 1, 16203. doi:10.1038/nmicrobiol.2016.203.

Loïez, C., Wallet, F., Pischedda, P., Renaux, E., Senneville, E., Mehdi, N., et al. (2007). First case of osteomyelitis caused by “Staphylococcus pettenkoferi.” *J. Clin. Microbiol.* 45, 1069–1071. doi:10.1128/JCM.02328-06.

Marsou, R., Bes, M., Boudouma, M., Brun, Y., Meugnier, H., Freney, J., et al. (1999). Distribution of Staphylococcus sciuri subspecies among human clinical specimens, and profile of antibiotic resistance. *Res. Microbiol.* 150, 531–541.

Pentima, M. (2010). Elizabethkingia, chryseobacterium and myroides species. *Antimicrobe*.

Solak, Y., Gul, E. E., Atalay, H., Genc, N., and Tonbul, H. Z. (2011). A rare human infection of Raoultella ornithinolytica in a diabetic foot lesion. *Ann. Saudi Med.* 31, 93–94. doi:10.4103/0256-4947.75794.

Song, Y., Liu, C., and Finegold, S. M. (2007). Peptoniphilus gorbachii sp. nov., Peptoniphilus olsenii sp. nov., and Anaerococcus murdochii sp. nov. isolated from clinical specimens of human origin. *J. Clin. Microbiol.* 45, 1746–1752. doi:10.1128/JCM.00213-07.

Stappers, M. H. T., Hagen, F., Reimnitz, P., Mouton, J. W., Meis, J. F., and Gyssens, I. C. (2015). Direct molecular versus culture-based assessment of Gram-positive cocci in biopsies of patients with major abscesses and diabetic foot infections. *Eur. J. Clin. Microbiol. Infect. Dis. Off. Publ. Eur. Soc. Clin. Microbiol.* 34, 1885–1892. doi:10.1007/s10096-015-2428-4.

Talay, S. R., Valentin-Weigand, P., Jerlström, P. G., Timmis, K. N., and Chhatwal, G. S. (1992). Fibronectin-binding protein of Streptococcus pyogenes: sequence of the binding domain involved in adherence of streptococci to epithelial cells. *Infect. Immun.* 60, 3837–3844.

Tong, H., Gao, X., and Dong, X. (2003). Streptococcus oligofermentans sp. nov., a novel oral isolate from caries-free humans. *Int. J. Syst. Evol. Microbiol.* 53, 1101–1104. doi:10.1099/ijs.0.02493-0.

Van Hoovels, L., Vankeerberghen, A., Boel, A., Van Vaerenbergh, K., and De Beenhouwer, H. (2006). First case of Staphylococcus pseudintermedius infection in a human. *J. Clin. Microbiol.* 44, 4609–4612. doi:10.1128/JCM.01308-06.
